# Supplementary material for: Comparative genomics provides new insights into the diversity, physiology, and sexuality of the only industrially exploited tremellomycete: Phaffia rhodozyma
Source: BMC Genomics. 2016 Nov 9;17:901. doi: 10.1186/s12864-016-3244-7 (PMC5103461; doi:10.1186/s12864-016-3244-7)
Supplement: Additional file 6: — List of orphan genes with links to PFAM (related to Additional file 1: Table S1). (ZIP 1428 kb) [file 12864_2016_3244_MOESM6_ESM.zip › PFAM_UNIQUE/G04031_P.html]

#                                                                            --- full sequence --- -------------- this domain -------------   hmm coord   ali coord   env coord
  
# target name        accession   tlen query name           accession   qlen   E-value  score  bias   #  of  c-Evalue  i-Evalue  score  bias  from    to  from    to  from    to  acc description of target
  
#------------------- ---------- ----- -------------------- ---------- ----- --------- ------ ----- --- --- --------- --------- ------ ----- ----- ----- ----- ----- ----- ----- ---- ---------------------
  
G04031\_P             -            554 Amidase              PF01425.16   441       3.6    5.0   6.0   1   2   0.00016      0.32    8.4   1.4   155   230   223   297   222   349 0.84 - Amidase  
G04031\_P             -            554 Amidase              PF01425.16   441       3.6    5.0   6.0   2   2      0.78   1.6e+03   -3.7   0.2   328   356   486   515   465   536 0.54 - Amidase  
G04031\_P             -            554 Baculo\_PP31          PF05311.6    272       1.3    6.9   7.1   1   1   0.00071       2.1    6.2   7.1   194   268   476   550   446   552 0.44 - Baculovirus 33KDa late protein (PP31)  
G04031\_P             -            554 BLVR                 PF06375.6    154      0.18   10.3   6.4   1   1    0.0011      0.43    9.1   6.4    76   118   492   541   448   548 0.56 - Bovine leukaemia virus receptor (BLVR)  
G04031\_P             -            554 BUD22                PF09073.5    432      0.31    8.7   9.2   1   1    0.0039      0.52    8.0   9.2   129   199   480   550   449   553 0.90 - BUD22  
G04031\_P             -            554 Draxin               PF15550.1    324       3.3    5.9   6.5   1   1     0.007         7    4.9   6.5    60   136   464   538   426   552 0.65 - Draxin  
G04031\_P             -            554 DUF1168              PF06658.7    142      0.17   10.1  16.4   1   1   0.00087      0.37    9.0  16.4    75   128   495   547   480   553 0.68 - Protein of unknown function (DUF1168)  
G04031\_P             -            554 DUF1682              PF07946.9    321       6.5    4.2   9.1   1   1     0.011       9.6    3.6   9.1   239   311   459   527   447   539 0.77 - Protein of unknown function (DUF1682)  
G04031\_P             -            554 DUF1777              PF08648.7    180       5.1    5.5   8.2   1   1     0.032       9.6    4.6   8.2    80   120   498   539   468   551 0.60 - Protein of unknown function (DUF1777)  
G04031\_P             -            554 DUF2011              PF09428.5    131       2.1    6.9   5.2   1   1    0.0041       3.5    6.2   5.2    83   113   497   525   449   541 0.52 - Fungal protein of unknown function (DUF2011)  
G04031\_P             -            554 DUF2992              PF11208.3    132       2.5    6.7   6.5   1   1     0.003       4.5    5.8   6.5    53    87   492   525   477   549 0.62 - Protein of unknown function (DUF2992)  
G04031\_P             -            554 DUF4391              PF14335.1    221     0.096   10.7   2.1   1   1   4.9e-05      0.15   10.1   2.1   128   209   443   527   428   532 0.78 - Domain of unknown function (DUF4391)  
G04031\_P             -            554 FLO\_LFY              PF01698.11   387       3.2    5.1   6.0   1   1    0.0087       4.3    4.7   6.0   169   211   489   540   431   553 0.47 - Floricaula / Leafy protein  
G04031\_P             -            554 GAGA\_bind            PF06217.7    301       3.1    6.3   7.7   1   1     0.014       5.8    5.4   7.7    98   184   451   539   421   552 0.37 - GAGA binding protein-like family  
G04031\_P             -            554 Med19                PF10278.4    178     0.027   12.8   4.9   1   1   8.8e-05     0.048   12.0   4.9   128   168   488   529   443   540 0.70 - Mediator of RNA pol II transcription subunit 19  
G04031\_P             -            554 MRP-S26              PF14943.1    170       5.2    5.3  10.3   1   1     0.013       7.9    4.7  10.3    49   125   465   541   446   550 0.83 - Mitochondrial ribosome subunit S26  
G04031\_P             -            554 Peptidase\_S49\_N      PF08496.5    155       2.3    6.6   5.2   1   1    0.0037       3.7    6.0   5.2    40    90   477   526   460   543 0.47 - Peptidase family S49 N-terminal  
G04031\_P             -            554 Raftlin              PF15250.1    457       1.6    5.8   6.2   1   1   0.00043       2.6    5.1   6.2   144   229   467   550   431   553 0.59 - Raftlin  
G04031\_P             -            554 RR\_TM4-6             PF06459.7    274       3.9    6.0   8.7   1   1     0.024       6.5    5.2   8.7    60   138   472   549   433   553 0.72 - Ryanodine Receptor TM 4-6  
G04031\_P             -            554 SART-1               PF03343.8    613       7.4    3.4  10.1   1   1     0.063        11    2.9  10.1   266   329   486   546   458   552 0.63 - SART-1 family  
G04031\_P             -            554 U79\_P34              PF03064.11   251       1.4    7.0   7.4   1   1    0.0037       2.2    6.4   7.4   166   211   496   544   454   553 0.55 - HSV U79 / HCMV P34  
G04031\_P             -            554 Zn\_clus              PF00172.13    40      0.29    9.8   2.4   1   1    0.0038      0.63    8.7   2.4     1    30   189   222   189   229 0.80 - Fungal Zn(2)-Cys(6) binuclear cluster domain
